# Dietary patterns and sleep disorders in Mexican adults from a National Health and Nutrition Survey

**DOI:** 10.1017/jns.2021.24

**Published:** 2021-05-11

**Authors:** Elsa B. Gaona-Pineda, Brenda Martinez-Tapia, Sonia Rodríguez-Ramírez, Selene Guerrero-Zúñiga, Rogelio Perez-Padilla, Teresa Shamah-Levy

**Affiliations:** 1 Center for Evaluation and Surveys Research, National Institute of Public Health, Av. Universidad 655, Santa Maria Ahuacatitlán, Cuernavaca, Morelos 62100, Mexico; 2 Center for Nutrition and Health Research, National Institute of Public Health, Cuernavaca, Morelos, Mexico; 3 Mexican National Institute of Respiratory Diseases, Mexico City, Mexico

**Keywords:** Dietary patterns, Health surveys, Mexico, Obstructive sleep apnoea, Sleep quality

## Abstract

Given the high prevalence of multiple non-communicable chronic diseases in Mexico, the aim of the present study was to assess the association between dietary patterns and sleep disorders in a national representative sample of 5076 Mexican adults (20–59 years) from the 2016 National Health and Nutrition Survey. Through a cross-sectional study, we used the Berlin sleep symptoms questionnaire to estimate the proportion of adults with insomnia, obstructive sleep apnoea (OSA) and other related problems such as daytime symptoms and inadequate sleep duration. Dietary data were collected through a seven-day semi-quantitative food frequency questionnaire, and dietary patterns were determined through cluster analysis. Associations between dietary patterns and sleep disorders were assessed by multivariate logistic regression models adjusted for age, sex, well-being, rural/urban area type, geographical region, tobacco use, physical activity level and energy intake. Three dietary patterns were identified: traditional (high in legumes and tortilla), industrialised (high in sugar-sweetened beverages, fast foods, and alcohol, coffee or tea) and mixed (high in meat, poultry, fruits and vegetables). Multivariate logistic regression showed that the industrialised pattern yielded higher odds for daytime symptoms (OR 1⋅49; 95 % CI 1⋅12, 1⋅99) and OSA (OR 1⋅63; 95 % CI 1⋅21, 2⋅19) compared with the traditional pattern. In conclusion, dietary patterns are associated with sleep disorders in Mexican adults. Further research is required to break the vicious cycle of poor-quality diet, sleep symptoms and health.

## Introduction

Epidemiological evidence suggests that sleep disorders are associated with adverse health conditions^(1)^. According to the National Sleep Foundation, a duration of 7–9 h of sleep are necessary for optimal health in adults (26–64 years of age)^(2)^. Less than 6 h is insufficient and has been associated with obesity^(3)^, hypertension^(4)^, type 2 diabetes^(5)^, cardiovascular disease (CVD)^(6)^ and all-cause mortality^(7)^.

Insomnia is one of the most common sleep disorders. It is characterised by difficulty initiating or maintaining sleep, premature awakening and non-restorative sleep^(8)^, accompanied by negative daytime consequences in social, occupational and behavioural performance as well as in other important life functions. These symptoms have been associated with mortality^(9,10)^, CVD^(11)^ and injury^(12)^.

Obstructive sleep apnoea (OSA), another prevalent sleep disorder, is defined by repetitive episodes of apnoea (cessation of inspiratory airflow during sleep which lasts ≥10 s) or hypopnoea (a reduction of ≥30 % in inspiratory airflow lasting ≥10 s, with an associated drop in oxygen saturation or arousal from sleep)^(13)^. This condition has been associated with many different forms of CVD including hypertension, stroke, heart failure, coronary artery disease and atrial fibrillation. Adults with OSA face not only an increased risk of developing comorbid CVD but also worse CVD-related outcomes^(14)^.

One of the primary symptoms associated with sleep disorders is excessive daytime sleepiness (EDS)^(15)^. This condition reduces performance in school^(16)^ or at work^(17,18)^ due to concentration, memory and mood problems^(19)^.

Given their role in adverse health outcomes, the causes of sleep disorders have been studied from various perspectives, including through diet. It has been reported that adequate sleep is positively associated with a healthy diet in children^(20)^, adolescents^(21–23)^ and adults^(24–26)^. Conversely, individuals with insufficient sleep duration are more likely to have lower-quality diets and adopt irregular meal patterns. They tend to consume more energy-rich foods, calories, fats, refined carbohydrates and snacks, and fewer proteins, fruits and vegetables^(27,28)^.

A study of Japanese workers found that a healthy dietary pattern (characterised by vegetables, mushrooms, potatoes, seaweed, soya products and eggs) was associated with a decreased risk of difficulty initiating sleep^(29)^. By contrast, a high intake of processed meat, red meat, snacks and take-out food was positively associated with the apnoea–hypopnoea index score after adjusting for demographic and lifestyle factors. Another study in Australia found that those who slept less time exhibited higher energy intake, especially from snacks and foods containing fat^(30)^.

Among middle-aged female Japanese workers, low protein intake was shown to be associated with difficulty initiating sleep, while high protein and low carbohydrate intake were associated with difficulty maintaining sleep^(31)^, and high carbohydrate intake with poor sleep quality^(32)^.

Few reports exist which document sleep disorders in Mexico, and those published only take place in Mexico City^(33,34)^ or with elderly participants^(35)^. The 2016 National Health and Nutrition Survey was the first study to reveal a high prevalence of multiple sleep disorders in the Mexican adult population: 28⋅4 % reported short sleep duration (<7 h), 18⋅8 % reported insomnia and 27⋅3 % showed high risk for OSA^(36)^. Furthermore, few studies have related diet with sleep disorders in Mexican adults. In one adolescent cohort, consumption of a healthy plant-based and lean protein dietary pattern was associated with an earlier bedtime, as assessed 2 years later^(37)^. In one cohort of female teachers, a fruit and vegetable-based dietary pattern was associated with greater sleep quality^(38)^. Given the documented relationship between sleep alterations and adverse health outcomes, the aim of the present study was to analyse the association between dietary patterns and sleep disorders in Mexican adults (age 20–59) based on data from a nationally representative survey.

## Methods

The 2016 National Health and Nutrition Survey (in Spanish, *ENSANUT-2016*) is a cross-sectional survey with a probabilistic and multistage sampling design. The data collected allows statistical inferences regarding health and nutrition in the Mexican population at the national, geographical region and urban/rural area levels^(39)^. We analysed a sample of adults aged 20–59 through information regarding sleep and dietary habits.

### Sleep disorders

The *ENSANUT-2016* surveyed a sample of the Mexican adult population aged ≥20 using the Berlin questionnaire on OSA risk, sleep duration, insomnia and EDS^(36)^. Average sleep duration over the 6 months before interview date was categorised as: <7 h, 7–9 h and >9 h^(40)^. Insomnia was defined as waking up prematurely or experiencing difficulty initiating or maintaining sleep ≥3–4 times per week within the last 3 weeks^(41)^. Daytime symptoms were defined as the perception of not feeling rested or feeling sleepy/tired during the day ≥3 d/week.

Participants with affirmative scores in two or more of the following symptom categories were classified as high-risk for OSA:^(42)^

Category 1. Nighttime symptoms were explored through five questions, with responses assigned the following values: snoring = 1 point; snoring loud or very loud = 1 point; snoring ≥3 d/week = 1 point; snoring in a way that has annoyed others = 1 point; and another person noticing the cessation of the respondent's breathing during sleep ≥3 d/week = 2 points. An affirmative result in this category was a score of ≥2 points.
Category 2. Daytime sleepiness was explored through three questions, with responses assigned the following values: the perception of non-restorative sleep ≥3 d/week = 1 point, daytime tiredness ≥3 d/week = 1 point and somnolence while driving = 1 point. An affirmative result in this category was a score of 2 points.
Category 3. An affirmative result in this category was considered as suffering from obesity or hypertension.

### Dietary intake data and dietary patterns

Dietary information was obtained from 6511 adults through a semi-quantitative food frequency questionnaire (SFFQ) on consumption during the 7 d before the survey. The SFFQ included a list of 140 foods and beverages. Consumption was estimated in grams based on the following parameters: number of days, times per day, portion size, number of portions consumed, edible portions (for meat, poultry, fruits and vegetables) and density (for beverages). Consumption data >4 sd from the mean by food or beverage item were considered implausible and those values were imputed with the average consumption by sex, urban/rural area and region (North, Centre, Mexico City and South). Participants with >7 food and beverage items with consumption imputations were excluded from analysis (*n* 8). Energy intake was estimated according to the nutrient database compiled by the National Institute of Public Health of Mexico (unpublished material). Energy intake requirements were estimated based on equations from the Institute of Medicine, considering sex, age, height and weight^(43)^ and the Mifflin-St Jeor equation was used to estimate resting metabolic rate^(44)^. Results showing the ratios of energy intake/energy requirement >3 sd from the average, by sex, were excluded from analysis, as were those which indicated an energy intake of less than half of the resting metabolic rate (*n* 161).

Foods and beverages were classified into twenty-six food groups according to their nutritional content. In addition, a separate food group included alcoholic and caffeinated beverages such as coffee and tea, due to their demonstrated relationship with sleep disorders^(45,46)^ (Table 1). Dietary patterns were determined through k-means cluster analysis using the standardised percentage of contribution to energy intake of each food group^(47)^. Two to five clusters were specified within the k-means analysis in order to evaluate which set of clusters discriminated better across groups, while still maintaining an adequate sample size within each cluster.

**Table 1.** Food group classifications

Food group	Foods and beverages included
Sugar-sweetened dairy beverages	Milk with added sugars, drinkable yoghurt and Mexican milk-based beverages (*atoles*) (homemade or commercially prepared)
Non-sugar-sweetened dairy beverages	Milk without added sugars (all types: whole, semi-skim, skim, lactose-free, etc.)
Sugar-sweetened non-dairy beverages	Water-based *atole*, soft drinks, natural and industrialised fruit juices and sugar-sweetened natural fruit water
Non-sugar-sweetened non-dairy beverages	Water, light soft drinks, non-sugar-sweetened natural fruit water and low-calorie beverages
Fruits	Strawberry, jicama, papaya, pineapple, grapefruit, grape, orange, mandarin orange, apple, pear, cantaloupe, watermelon, peach, banana, guava and mango
Vegetables	Cucumber, carrot, tomato, chayote, leafy greens (chard, spinach, pigweed), cabbage, avocado, lettuce, broccoli, carrot, maize, zucchini, chilli pepper (all types) and onion (other root vegetables were not included)
Dairy products (non-beverage)	Yoghurt (natural, light or fruit-added) and cheese of all types
Legumes	Beans, lentils, chickpeas, broad beans and string beans (fresh or canned)
Cereal-based salty dishes	Rice and pasta
Corn-based savory dishes	Corn-based fried and non-fried Mexican dishes: *sopes*, *quesadillas*, *enchiladas*, *tacos*, *tlacoyos*, *gorditas*, *pozole*, *tamales*
Fast food	Hamburgers, pizza, hot dogs and sandwiches
Eggs	Boiled eggs, fried eggs and egg-battered vegetables
Meat, poultry and fish	Poultry, beef, pork, fish and shellfish
Processed meats	Processed ham and sausage
Pastries and cookies	Donuts, cakes, pies, cupcakes, cookies and pastries
Candies	Chocolate, candies, marshmallows and lollipops
Desserts	Fried plantains, gelatins, ice cream, frozen juice bars, sorbet, dried fruit and candied fruit
Salty snacks	Popcorn and chips (all kinds)
Nuts and seeds	Peanuts, tree nuts and fried broad beans
Added fats	Butter, lard, vegetable oil, margarine, mayonnaise and cream
Tortillas	Corn tortilla (homemade or commercially prepared). The corn tortilla is a staple food in traditional Mexican cooking; it is a thin, circular unleavened flatbread made of corn flour
Soups	Instant soups, vegetable soups and broths (beef, chicken and vegetable)
Ready-to-eat cereals	Ready-to-eat cereals (all kinds): basic, fibre-added, multi-ingredient, sugar-sweetened or flavoured
Breads	White bread, whole-wheat bread and crackers
Potatoes	Boiled or fried potatoes
Alcoholic beverages, coffee and tea	Coffee, tea and alcoholic beverages (all kinds)

### Covariables

The following variables were drawn from a health items questionnaire: lifetime tobacco use (>100, ≤100 or 0 cigarettes)^(48)^ and previous diagnosis of hypertension. The variables of age, sex and well-being index (WBI) were drawn from a socio-demographic questionnaire. WBI was estimated through principal component analysis based on housing characteristics (roof, wall and flooring materials; drainage; water provision) and material possessions (television, computer, radio, telephone, cable television, refrigerator, microwave, stove, boiler, washing machine). The percentage of variance explained by the first component was 40 %. WBI was categorised into tertiles, with tertile 1 being low and 3 being high.

Overweight and obesity were determined by body mass index (BMI) (weight in kilograms/height in meters^2^). BMI was categorised according to the cut-off points established by the World Health Organization (WHO)^(49)^. Also in line with WHO recommendations, physical activity was estimated using the International Physical Activity Questionnaire (IPAQ) validated in the Mexican adult population^(50)^.

### Statistical analysis

We estimated proportions and means of the categorical and continuous variables, respectively, with 95 % confidence intervals (95 % CIs). Comparison among groups was performed through linear, logit or quantile regression, depending on the variable type. The Bonferroni method was applied to adjust *P*-value^(51)^. Logistic regression models were used to assess the association between dietary patterns and each sleep disorder, and models were adjusted by age, sex, WBI, rural/urban area, geographical region, tobacco use, physical activity level, total energy intake and BMI (with the exception of the OSA logistic regression model, since obesity defined as BMI ≥30 kg/m^2^ is included in the classification criteria for high-risk OSA). All logistic regression models were stratified by sex. Sensitivity analyses were performed accounting for use of sedative medications. All statistical analyses were performed using STATA 14.0 with survey commands (SVY).

## Results

We analysed data from 5076 adults who answered the sleep questionnaire described earlier and who indicated biologically plausible dietary intakes. The sample represented 59 251 709 Mexican adults 20–59 years old, of whom 57⋅13 % were in the 20–39 age group. A higher proportion of men had smoked >100 cigarettes in their lifetime, as compared with women (48⋅76 %; 95 % CI 45⋅06, 52⋅48 *vs.* 14⋅87 %; 95 % CI 12⋅50, 17⋅59). Conversely, women showed a greater prevalence of obesity, as well as hypertension, insomnia and daytime symptoms. Insomnia was reported by 16⋅57 % of the study population, and daytime symptoms by 19⋅48 %. OSA risk was identified in 26⋅22 % of the population (Table 2).

**Table 2.** Descriptive characteristics of Mexican adult study participants, by sex

Characteristic	Male				Female					
	*n*	*N*^a^	%	95 % CI	*n*	*N*^a^	%	95 % CI	National %	95 % CI
Age group (years)										
20–39	848	16 660⋅06	58⋅74	55⋅05, 62⋅34	1728	17 188⋅69	55⋅64	52⋅68, 58⋅57	57⋅13	54⋅58, 59⋅64
40–59	888	11 701⋅44	41⋅26	37⋅66, 44⋅95	1612	13 701⋅52	44⋅36	41⋅43, 47⋅32	42⋅87	40⋅36, 45⋅42
Area type										
Rural	928	7659⋅29	27⋅01	23⋅45, 30⋅88	1665	7348⋅91	23⋅79	20⋅64, 27⋅25	25⋅33	22⋅39, 28⋅51
Urban	808	20 702⋅21	72⋅99	69⋅12, 76⋅55	1675	23 541⋅30	76⋅21	72⋅75, 79⋅36	74⋅67	71⋅49, 77⋅61
Geographical region										
North	379	5907⋅33	20⋅83	17⋅49, 24⋅62	736	6510⋅70	21⋅08	16⋅99, 25⋅84	20⋅96	17⋅86, 24⋅44
Centre	576	9734⋅7	34⋅32	29⋅99, 38⋅93	1135	10 285⋅98	33⋅30	29⋅42, 37⋅41	33⋅79	30⋅29, 37⋅47
Mexico City	182	4511⋅95	15⋅91	12⋅53, 20⋅00	375	5040⋅54	16⋅32	12⋅21, 21⋅47	16⋅12	13⋅10, 19⋅69
South	599	8207⋅52	28⋅94	25⋅02, 33⋅2	1094	9052⋅99	29⋅31	25⋅08, 33⋅92	29⋅13	25⋅56, 32⋅98
Well-being index										
Tertile 1	877	9762⋅14	34⋅42	30⋅36, 38⋅72	1557	9935⋅07	32⋅16	28⋅66, 35⋅88	33⋅24	30⋅19, 36⋅45
Tertile 2	521	9577⋅38	33⋅77	30⋅54, 37⋅16	1119	10 168⋅98	32⋅92	29⋅62, 36⋅4	33⋅33	30⋅9, 35⋅85
Tertile 3	338	9021⋅99	31⋅81	27⋅99, 35⋅9	664	10 786⋅16	34⋅92	30⋅41, 39⋅71	33⋅43	30⋅37, 36⋅64
Tobacco use (lifelong)										
>100 cigarettes	762	13 648⋅72	48⋅76	45⋅06, 52⋅48	359	4562⋅53	14⋅87	12⋅50, 17⋅59	31⋅03	28⋅51, 33⋅68
≤100 cigarettes	483	7458⋅79	26⋅65	23⋅76, 29⋅75	815	9171⋅53	29⋅88	26⋅33, 33⋅70	28⋅34	25⋅94, 30⋅87
No cigarettes	473	6882⋅29	24⋅59	21⋅39, 28⋅09	2144	16 957⋅25	55⋅25	51⋅08, 59⋅35	40⋅63	37⋅66, 43⋅66
BMI (kg/m^2^)^b^	1709	27 770⋅66	27⋅74	27⋅33, 28⋅15	3295	30 400⋅02	28⋅90	28⋅53, 29⋅26	28⋅34	28⋅05, 28⋅64
BMI category										
Normal	498	8386⋅10	30⋅20	26⋅81, 33⋅81	747	7693⋅31	25⋅31	22⋅73, 28⋅07	27⋅64	25⋅54, 29⋅85
Overweight	709	11 636⋅25	41⋅90	38⋅13, 45⋅77	1209	10 810⋅25	35⋅56	32⋅65, 38⋅58	38⋅59	36⋅2, 41⋅03
Obesity	502	7748⋅31	27⋅90	24⋅58, 31⋅48	1339	11 896⋅46	39⋅13	36⋅10, 42⋅26	33⋅77	31⋅33, 36⋅3
Hypertension (yes)	145	2154⋅65	7⋅69	5⋅90, 9⋅97	413	3801⋅92	12⋅37	10⋅38, 14⋅68	10⋅14	8⋅68, 11⋅82
Physical activity level										
Inactive	176	3211⋅66	11⋅71	9⋅45, 14⋅42	492	4123⋅02	13⋅81	11⋅53, 16⋅45	12⋅80	11⋅06, 14⋅78
Moderately active	128	1992⋅58	7⋅27	5⋅04, 10⋅36	362	3490⋅50	11⋅69	9⋅94, 13⋅69	9⋅57	8⋅22, 11⋅12
Active	1380	22 219⋅67	81⋅02	77⋅29, 84⋅27	2381	22 251⋅03	74⋅51	71⋅52, 77⋅28	77⋅63	75⋅38, 79⋅73
Sleep duration (hours)										
7–9	1198	18 854⋅29	66⋅48	62⋅55, 70⋅19	2410	21 096⋅55	68⋅30	65⋅33, 71⋅11	67⋅43	64⋅96, 69⋅79
<7	453	8005⋅46	28⋅23	25⋅02, 31⋅66	704	7505⋅72	24⋅30	22⋅10, 26⋅64	26⋅18	24⋅14, 28⋅32
>9	85	1501⋅76	5⋅30	3⋅71, 7⋅51	226	2287⋅93	7⋅41	5⋅48, 9⋅94	6⋅40	5⋅10, 8⋅00
Insomnia (yes)	223	3592⋅26	12⋅67	10⋅51, 15⋅19	692	6224⋅75	20⋅15	17⋅74, 22⋅80	16⋅57	14⋅94, 18⋅33
Daytime symptoms (yes)	238	4100⋅08	14⋅46	11⋅99, 17⋅33	747	7441⋅65	24⋅09	21⋅22, 27⋅21	19⋅48	17⋅53, 21⋅59
Obstructive sleep apnoea (yes)	438	7244⋅71	25⋅54	22⋅50, 28⋅85	853	8290⋅63	26⋅84	23⋅7, 30⋅23	26⋅22	23⋅88, 28⋅70

a *N* (thousands) = Expansion of the sample expressed per 1000 individuals, considering survey sample weights.

b Mean and 95 % CI.

### Dietary patterns

We identified three dietary patterns: traditional, industrialised and mixed. The traditional pattern yielded the highest mean intake of tortilla (42⋅75 %) and legumes (4⋅42 %). The industrialised pattern yielded the highest intake of sugar-sweetened non-dairy beverages (11⋅01 %), alcoholic beverages, coffee or tea (8⋅45 %), bakery products and cookies (7⋅37 %), fast foods (5⋅79 %), breads (5⋅58 %) and candies (1⋅32 %). The mixed pattern yielded the highest intakes of fruits (9⋅48 %), meat and poultry (8⋅51 %), vegetables (6⋅19 %), non-sugar-sweetened dairy beverages (4⋅00 %), sugar-sweetened dairy beverages (3⋅71 %) and cereal-based salty recipes (3⋅34 %) (Table 3).

**Table 3.** Percentage of energy intake contribution of each food group, by dietary pattern

Food group	Traditional dietary pattern *n* 1969, *N* 16 847⋅7		Industrialised dietary pattern *n* 1683, *N* 24 334⋅2		Mixed dietary pattern *n* 1424, *N* 18 069⋅8	
	Mean	95 % CI	Mean	95 % CI	Mean	95 % CI
Tortilla^a^	42⋅75*^,^**	41⋅34, 44⋅17	16⋅53	15⋅71, 17⋅35	17⋅89	16⋅81, 18⋅97
Sugar-sweetened non-dairy beverages	6⋅52	6⋅01, 7⋅03	11⋅01*	10⋅26, 11⋅76	5⋅27**^,^***	4⋅78, 5⋅77
Corn-based savory dishes	7⋅00	6⋅09, 7⋅92	7⋅45	6⋅69, 8⋅21	4⋅91**^,^***	4⋅27, 5⋅55
Meat, poultry and fish	3⋅91	3⋅54, 4⋅27	7⋅00*	6⋅48, 7⋅52	8⋅51**^,^***	7⋅86, 9⋅17
Fruits	4⋅03	3⋅69, 4⋅38	3⋅79	3⋅53, 4⋅06	9⋅48**^,^***	8⋅93, 10⋅03
Pastries and cookies	4⋅54	4⋅05, 5⋅03	7⋅37*	6⋅77, 7⋅98	4⋅93***	4⋅44, 5⋅43
Alcoholic beverages, coffee and tea	4⋅88	4⋅28, 5⋅47	8⋅45*	7⋅47, 9⋅43	3⋅22**^,^***	2⋅76, 3⋅69
Eggs	3⋅85	3⋅43, 4⋅26	4⋅07	3⋅72, 4⋅42	3⋅44	3⋅13, 3⋅75
Breads	2⋅12	1⋅8, 2⋅44	5⋅58*	4⋅86, 6⋅29	3⋅62**^,^***	3⋅08, 4⋅16
Vegetables	2⋅30	2⋅12, 2⋅49	2⋅42	2⋅22, 2⋅62	6⋅19**^,^***	5⋅65, 6⋅73
Fast food	1⋅04	0⋅85, 1⋅23	5⋅79*	5⋅25, 6⋅32	2⋅79**^,^***	2⋅36, 3⋅22
Dairy products (non-beverage)	1⋅87	1⋅59, 2⋅15	2⋅65*	2⋅31, 2⋅99	4⋅87**^,^***	4⋅42, 5⋅33
Legumes	4⋅42	4⋅02, 4⋅82	2⋅08*	1⋅89, 2⋅27	2⋅64**^,^***	2⋅41, 2⋅87
Cereal-based salty dishes	2⋅65	2⋅39, 2⋅9	2⋅21*	2⋅04, 2⋅38	3⋅34**^,^***	3⋅08, 3⋅61
Non-sugar-sweetened dairy beverages	1⋅44	1⋅14, 1⋅73	1⋅89	1⋅63, 2⋅14	4⋅00**^,^***	3⋅39, 4⋅62
Sugar-sweetened dairy beverages	1⋅27	0⋅97, 1⋅57	1⋅66	1⋅38, 1⋅93	3⋅71**^,^***	3⋅04, 4⋅38
Processed meats	0⋅96	0⋅82, 1⋅09	1⋅82*	1⋅63, 2⋅02	1⋅32**^,^***	1⋅09, 1⋅55
Desserts	0⋅52	0⋅43, 0⋅62	1⋅13*	0⋅99, 1⋅27	1⋅91**^,^***	1⋅68, 2⋅13
Potatoes	1⋅01	0⋅85, 1⋅17	0⋅98	0⋅85, 1⋅11	1⋅21	1⋅04, 1⋅39
Non-sugar-sweetened non-dairy beverages	0⋅66	0⋅53, 0⋅79	0⋅69	0⋅6, 0⋅77	1⋅44**^,^***	1⋅21, 1⋅67
Salty snacks	0⋅47	0⋅34, 0⋅59	1⋅59*	1⋅38, 1⋅8	0⋅58***	0⋅45, 0⋅72
Soups	0⋅57	0⋅5, 0⋅64	0⋅64	0⋅58, 0⋅7	1⋅53**^,^***	1⋅36, 1⋅7
Added fats	0⋅56*^,^**	0⋅31, 0⋅81	0⋅99	0⋅84, 1⋅14	0⋅93	0⋅76, 1⋅1
Candies	0⋅26	0⋅19, 0⋅33	1⋅32*	1⋅15, 1⋅48	0⋅58**^,^***	0⋅48, 0⋅67
Ready-to-eat cereals	0⋅12	0⋅09, 0⋅16	0⋅31*	0⋅24, 0⋅38	1⋅13**^,^***	0⋅95, 1⋅32
Nuts and seeds	0⋅29*^,^**	0⋅21, 0⋅38	0⋅58	0⋅44, 0⋅73	0⋅53	0⋅37, 0⋅69

a The corn tortilla is a staple food in traditional Mexican cooking; it is a thin, circular unleavened flatbread made of corn flour.

* Significant difference between the traditional and industrialised patterns (*P* < 0⋅017).

** Significant difference between the traditional and mixed patterns (*P* < 0⋅017).

*** Significant difference between the industrialised and mixed patterns (*P* < 0⋅017).

Table 4 shows that the traditional pattern had the lowest median protein intake. The industrialised pattern had the highest median intake of lipids, as well as of all specific varieties of fats and sugars, and the lowest median fibre intake. The mixed pattern showed the lowest median caffeine intake.

**Table 4.** Nutritional characteristics, by dietary pattern

Energy and nutrients	Traditional dietary pattern		Industrialised dietary pattern		Mixed dietary pattern	
	Median	p25, p75	Median	p25, p75	Median	p25, p75
Energy (kcal/d)	1802⋅89	1355⋅42, 2391⋅62	2115⋅07*^,^***	1618⋅74, 2806⋅90	1681⋅58	1293⋅82, 2220⋅36
Carbohydrates (g/d)	300⋅93	227⋅74, 380⋅08	298⋅05	223⋅46, 389⋅89	237⋅95**^,^***	188⋅42, 313⋅68
Lipids (g/d)	44⋅54	31⋅78, 63⋅44	66⋅66*	49⋅40, 90⋅37	55⋅60**^,^***	38⋅88, 78⋅29
Proteins (g/d)	55⋅59*^,^**	40⋅19, 74⋅20	65⋅57	50⋅18, 88⋅38	63⋅09	44⋅52, 81⋅81
Fibers (g/d)	30⋅61	22⋅04, 40⋅42	22⋅25*	15⋅83, 30⋅38	25⋅91**^,^***	18⋅37, 34⋅20
Sugars (g/d)	69⋅70	45⋅62, 102⋅49	121⋅68*	81⋅91, 184⋅43	95⋅33**^,^***	68⋅79, 129⋅95
Caffeine (mg/d)	85⋅25	15⋅86, 221⋅80	101⋅73	38⋅43, 225⋅32	33⋅51**^,^***	7⋅80, 148⋅15
Alcohol (g/d)	0⋅00	0⋅00, 0⋅002	0⋅002	0⋅00, 17⋅48	0⋅00	0⋅00, 0⋅01
Saturated fats (g/d)	14⋅69	9⋅29, 20⋅91	24⋅08*	17⋅44, 33⋅40	20⋅31**^,^***	13⋅52, 29⋅71
Monounsaturated fats (g/d)	13⋅81	9⋅54, 19⋅63	22⋅57*	16⋅45, 29⋅00	18⋅09**^,^***	12⋅01, 26⋅74
Polyunsaturated fats (g/d)	12⋅87	8⋅75, 18⋅41	15⋅68*	11⋅30, 23⋅04	11⋅62**^,^***	7⋅98, 17⋅33
Trans fats (g/d)	0⋅52	0⋅20, 1⋅32	1⋅14*	0⋅57, 2⋅24	1⋅13**^,^***	0⋅47, 2⋅71

* Significant difference between the traditional and industrialised patterns (*P* < 0⋅017).

** Significant difference between the traditional and mixed patterns (*P* < 0⋅017).

*** Significant difference between the industrialised and mixed patterns (*P* < 0⋅017).

Table 5 shows the distribution of participant characteristics by dietary pattern. Adults with the industrialised dietary pattern exhibited the highest energy intake and included the highest proportion of younger adults (aged 20–39), smokers (>100 cigarettes) and individuals at high risk for OSA, while showing the lowest proportion of ‘active’ individuals by physical activity level. Adults with the traditional pattern included the highest proportion of individuals living in rural areas and in southern Mexico, and of individuals in WBI tertile 1 (low). A higher proportion of adults with the traditional pattern reported sleeping 7–9 h/d and suffering less daytime symptoms, compared with the industrialised pattern.

**Table 5.** Descriptive characteristics of Mexican adult study participants, by dietary pattern

Characteristic	Traditional dietary pattern			Industrialised dietary pattern			Mixed dietary pattern		
	*n*	%	95 % CI	*n*	%	95 % CI	*n*	%	95 % CI
Sex									
Male	745	52⋅10	48⋅44, 55⋅74	688	56⋅11	52⋅54, 59⋅61	303	32⋅82**^,^***	28⋅13, 37⋅89
Female	1224	47⋅90	44⋅26, 51⋅56	995	43⋅89	40⋅39, 47⋅46	1121	67⋅18*^,^***	62⋅11, 71⋅87
Age group (years)									
20–39	921	50⋅57	46⋅53, 54⋅6	993	64⋅48*^,^***	60⋅67, 68⋅12	662	53⋅33	48⋅58, 58⋅02
40–59	1048	49⋅43	45⋅4, 53⋅47	690	35⋅52*^,^***	31⋅88, 39⋅33	762	46⋅67	41⋅98, 51⋅42
Area type									
Rural	1352	44⋅88*^,^**	39⋅42, 50⋅46	682	17⋅56	14⋅37, 21⋅29	559	17⋅56	14⋅07, 21⋅71
Urban	617	55⋅12*^,^***	49⋅54, 60⋅58	1001	82⋅44	78⋅71, 85⋅63	865	82⋅44	78⋅29, 85⋅93
Geographical region									
North	364	13⋅82*	10⋅13, 18⋅57	510	27⋅03	23⋅28, 31⋅13	241	19⋅44	13⋅65, 26⋅93
Centre	652	33⋅80	28⋅83, 39⋅16	540	34⋅27	29⋅8, 39⋅05	519	33⋅12	28⋅11, 38⋅55
Mexico City	100	8⋅43*^,^**	5⋅93, 11⋅86	248	18⋅80	15⋅43, 22⋅72	209	19⋅68	14⋅49, 26⋅17
South	853	43⋅95*^,^**	38⋅33, 49⋅72	385	19⋅90	16⋅31, 24⋅04	455	27⋅75	22⋅74, 33⋅39
Well-being index									
Tertile 1	1334	58⋅05*^,^**	53⋅02, 62⋅92	616	24⋅16	20⋅95, 27⋅7	484	22⋅34	18⋅77, 26⋅37
Tertile 2	496	31⋅01	26⋅8, 35⋅56	622	35⋅09	31⋅2, 39⋅19	522	33⋅11	28⋅65, 37⋅89
Tertile 3	139	10⋅94*^,^**	8⋅484, 13⋅99	445	40⋅75	36⋅42, 45⋅23	418	44⋅55	38⋅56, 50⋅7
Tobacco use (lifetime)									
>100 cigarettes	358	25⋅60	22⋅18, 29⋅35	519	42⋅61*^,^***	38⋅52, 46⋅8	244	20⋅53	16⋅43, 25⋅35
≤100 cigarettes	473	27⋅06	23⋅43, 31⋅03	450	26⋅36	22⋅87, 30⋅18	375	32⋅18	27⋅38, 37⋅38
None	1125	47⋅34	42⋅93, 51⋅79	700	31⋅03*^,^***	27⋅44, 34⋅87	792	47⋅29	41⋅47, 53⋅19
BMI^a^	1947	27⋅87*	27⋅43, 28⋅31	1657	28⋅74	28⋅23, 29⋅25	1400	28⋅26	27⋅75, 28⋅77
BMI category									
Normal	523	28⋅64	25⋅07, 32⋅5	404	25⋅70	22⋅26, 29⋅48	318	29⋅30	24⋅94, 34⋅06
Overweight	782	43⋅16	38⋅99, 47⋅43	600	37⋅21	33⋅32, 41⋅27	536	36⋅12	31⋅75, 40⋅73
Obese	642	28⋅20*	24⋅46, 32⋅27	653	37⋅09	33⋅1, 41⋅26	546	34⋅58	30⋅65, 38⋅74
Hypertension (yes)	204	9⋅36	7⋅164, 12⋅14	177	10⋅61	8⋅398, 13⋅31	177	10⋅24	7⋅793, 13⋅33
Physical activity level									
Inactive	236	12⋅36	9⋅54, 15⋅86	254	16⋅00	13⋅05, 19⋅46	178	8⋅98***	6⋅956, 11⋅51
Moderately active	161	7⋅05	5⋅33, 9⋅259	180	10⋅51	8⋅322, 13⋅18	149	10⋅67	8⋅394, 13⋅48
Active	1508	80⋅60	76⋅61, 84⋅05	1204	73⋅50*^,^***	69⋅66, 77⋅01	1049	80⋅35	76⋅94, 83⋅36
Sleep duration (hours)									
7–9	1472	73⋅12*	69⋅19, 76⋅73	1130	62⋅52	58⋅76, 66⋅13	1006	68⋅72	63⋅29, 73⋅68
<7	377	20⋅98*	17⋅86, 24⋅48	456	31⋅30	27⋅76, 35⋅08	324	24⋅13	20⋅28, 28⋅44
>9	120	5⋅90	4⋅14, 8⋅33	97	6⋅18	4⋅47, 8⋅48	94	7⋅15	4⋅53, 11⋅11
Insomnia (yes)	292	15⋅85	13⋅2, 18⋅92	356	17⋅26	14⋅78, 20⋅06	267	16⋅31	13⋅42, 19⋅68
Daytime symptoms (yes)	337	15⋅54*	13⋅07, 18⋅39	387	22⋅66	19⋅45, 26⋅22	261	18⋅86	15⋅58, 22⋅66
Obstructive sleep apnoea (yes)	445	21⋅68	18⋅27, 25⋅53	496	30⋅65*^,^***	27⋅27, 34⋅24	350	24⋅49	20⋅94, 28⋅41

a Mean and 95 % CI.

* Significant difference between the traditional and industrialised patterns (*P* < 0⋅017).

** Significant difference between the traditional and mixed patterns (*P* < 0⋅017).

*** Significant difference between the industrialised and mixed patterns (*P* < 0⋅017).

### Associations between dietary patterns and sleep disorders

Table 6 describes the associations between dietary patterns and insomnia, daytime symptoms, <7 h sleep duration and high risk for OSA (all at the national level and stratified by sex). Adults with the industrialised dietary pattern had higher odds of experiencing daytime symptoms than those with the traditional pattern (OR 1⋅49; *P* = 0⋅007), mainly due to an outsized effect in women. Furthermore, adults with the industrialised dietary pattern showed significantly higher odds of being at high risk for OSA (OR 1⋅63; *P* = 0⋅001) than those with the traditional dietary pattern, driven by a significant association in men. For women, the industrialised dietary pattern increased odds for sleep duration <7 h (OR 1⋅63; *P* = 0⋅04). Insomnia was not associated with any dietary pattern in this sample. Complete logistic regression models are given in Supplementary Table S1 of Supplementary material.

**Table 6.** Associations among dietary patterns and sleep disorders in Mexican adult study participants (Odds ratio and 95 % confidence interval)

	Males			Females			National		
	OR	95 % CI	*P*-value	OR	95 % CI	*P*-value	OR	95 % CI	*P*-value
Insomnia^a^									
Dietary pattern									
Traditional	1⋅00						1⋅00		
Industrialised	1⋅05	0⋅58, 1⋅90	0⋅878	1⋅11	0⋅75, 1⋅63	0⋅601	1⋅08	0⋅77, 1⋅53	0⋅654
Mixed	0⋅92	0⋅52, 1⋅62	0⋅768	0⋅87	0⋅60, 1⋅24	0⋅432	0⋅87	0⋅63, 1⋅19	0⋅387
Sex									
Male							1⋅00		
Female							1⋅65	1⋅18, 2⋅3	0⋅003
Daytime symptoms^a^									
Dietary pattern									
Traditional	1⋅00			1⋅00			1⋅00		
Industrialised	1⋅42	0⋅82, 2⋅46	0⋅208	1⋅51	1⋅04, 2⋅19	0⋅032	1⋅49	1⋅12, 1⋅99	0⋅007
Mixed	1⋅45	0⋅78, 2⋅70	0⋅239	0⋅75	0⋅51, 1⋅08	0⋅121	0⋅93	0⋅66, 1⋅3	0⋅665
Sex									
Male							1⋅00		
Female							2⋅17	1⋅51, 3⋅13	<0⋅0001
Sleep duration <7 h^a^									
Dietary pattern									
Traditional	1⋅00			1⋅00			1⋅00		
Industrialised	1⋅06	0⋅68, 1⋅67	0⋅797	1⋅62	1⋅02, 2⋅58	0⋅040	1⋅32	0⋅96, 1⋅8	0⋅088
Mixed	0⋅76	0⋅441, 1⋅3	0⋅323	1⋅18	0⋅80, 1⋅76	0⋅405	0⋅98	0⋅71, 1⋅36	0⋅906
Sex									
Male							1⋅00		
Female							0⋅84	0⋅65, 1⋅08	0⋅176
Obstructive sleep apnoea^b^									
Dietary pattern									
Traditional	1⋅00			1⋅00			1⋅00		
Industrialised	1⋅86	1⋅24, 2⋅81	0⋅003	1⋅41	0⋅9, 2⋅14	0⋅113	1⋅63	1⋅21, 2⋅19	0⋅001
Mixed	1⋅31	0⋅82, 2⋅11	0⋅253	1⋅00	0⋅65, 1⋅54	0⋅992	1⋅14	0⋅83, 1⋅55	0⋅424
Sex									
Male							1⋅00		
Female							1⋅25	0⋅93, 1⋅68	0⋅14

a Adjusted by age, sex, body mass index, rural/urban area type, geographical region, physical activity level, lifetime tobacco use, tertiles of well-being index and total energy intake (kcal).

b Adjusted by age, sex, rural/urban area type, geographical region, physical activity level, lifetime tobacco use, tertiles of well-being index and total energy intake (kcal).

In the present study sample, 131 subjects reported the use of sedative medications (2⋅62 %; 95 %CI 1⋅98, 3⋅44); logistic regression models were run excluding these participants and subsequent estimations remained unchanged (data not shown).

## Discussion

We identified three dietary patterns in our study population: industrialised, traditional and mixed. The industrialised pattern was defined by a high intake of food groups containing refined sugars, saturated fats and stimulant components such as caffeine and alcohol. The present results show that Mexican adults with an industrialised dietary pattern are significantly more likely to experience daytime symptoms and OSA than those with a traditional dietary pattern.

Although these associations differed in their level of significance by sex, it is important to note that the direction of the association is the same for each sex. This could be explained knowing that the most frequent sleep disorders in women are mainly daytime sleepiness^(52)^ and insufficient sleep time, while in men, OSA is more frequent^(53)^. The lack of statistical significance for daytime symptoms and sleep duration (*P* < 0⋅05) in men could be due in part to our sample size. Notably, the industrialised pattern remained the pattern most associated with sleep disorders in both sexes.

Similar results have been obtained in other contexts. For example, a study in China revealed that the traditional dietary pattern – characterised by high intakes of wheat and other staple foods – was associated with a decreased prevalence of daytime symptoms (OR 0⋅94; 95 % CI 0⋅89, 0⋅99; *P* = 0⋅004) and sleep disorders^(28)^. Likewise, when analysing the association between the Mediterranean diet and sleep quality, St-Onge *et al.* found that increased caloric intake was linked to daytime sleepiness. The high caloric intake observed by these authors may have been related to the consumption of more industrialised foods, in contrast with the Mediterranean diet^(54)^. Although the so-called traditional dietary patterns in these studies are not comparable in cultural terms, they share certain characteristics such as lower meat consumption and higher cereal consumption, as well as lower energy intake as compared with their counterparts.

A study in peri-menopausal Mexican women showed that those in the least-healthy dietary pattern quartile of fruit and vegetable intake, as compared with the healthiest, were 21 % more likely to report poor sleep quality at follow-up (95 % CI 1⋅06, 1⋅42). Those within the highest quartiles of the ‘modern Mexican’ pattern (tortillas and sodas, low in fibre and dairy) were 23 % more likely to report poor sleep quality as compared with the lowest quartiles (95 % CI 1⋅06, 1⋅43)^(38)^.

Finally, a study of female Japanese workers indicated that an increased intake of candies and noodles – as opposed to fish and vegetables – was associated with poor sleep quality. This may have been linked to a high carbohydrate intake^(32)^.

A relationship between high alcohol intake and daytime symptoms was reported by Lohsoonthorn *et al.*, who found that college students consuming ≥20 drinks/month had an OR of 3⋅10 (95 % CI 1⋅72, 5⋅59) for daytime symptoms^(55)^. Furthermore, when analysing the effect of alcohol on sleep physiology in non-alcoholic adults, Roehrs and Roth observed that alcohol consumption altered sleep stages and reduced rapid eye movement sleep time^(56)^. This may be explained by the effects of alcohol on hormonal functions related to the circadian rhythm; for instance, alcohol intake reduces the number of growth hormones. It has been documented that alcohol consumption affects specific neurotransmitters – e.g., gamma-aminobutyric acid (GABA) and acetylcholine – involved in the sleep–wake cycle. Reduced sleep quality hampers daytime alertness. A similar mechanism has been reported regarding the effect of caffeine on sleep disruption and perceived sleep quality, affecting the function of the hormone adenosine in the regulation of sleep–wake cycles^(57)^.

One study in young Mexican-American adults suggested that alcohol-use disorders are significantly associated with poorer sleep quality and that substance-use disorders may affect different aspects of sleep than do anxiety and depressive disorders^(58)^. In addition, a study on Mexican adolescents documented that males with a relatively older age, non-students, with more screen time, and having smoked cigarettes at any point, were associated with a later weekday sleep midpoint^(37)^.

In regards to the association between dietary intake and OSA, the present results are compatible with those of Vasquez *et al.*, where patients with OSA and a severe respiratory disturbance index score (RDI ≥ 50) showed higher intakes of cholesterol, saturated fats and trans fats^(59)^. These types of nutrients are key components of the food groups characterising the industrialised dietary pattern. Similar results were reported in Multi-Ethnic Study of Atherosclerosis, which found that adults with moderate to severe OSA had higher dietary quality scores. This association was also found with red/processed meats and whole-grain components scores^(60)^, and 6–9 % of this relationship was explained by a reduction in N3 sleep (slow-wave sleep) duration in individuals with OSA. In the present study, the industrialised pattern was characterised by a higher consumption of processed meats in contrast with the traditional and mixed patterns, as well as showing lower tortilla consumption than the traditional pattern.

Limited information is available on the biological mechanism driving the association between dietary patterns and OSA. Barceló *et al.* proposed a biological explanation, with the results of their study indicating higher levels of neuropeptide Y and leptin in OSA patients. Neuropeptide Y is an appetite-stimulating peptide, and the role of leptin in regulating energy balance is well-established^(61)^. In a laboratory-based randomised crossover study of adults aged 30–45, Shechter reported that subjects with lower N3 sleep increased fat and carbohydrate intake^(62)^. Frank *et al.* proposed a relationship between sleep quality and sleep disorders, and poor dietary quality^(63)^. In addition, a meta-analysis of randomised clinical trials showed that sleep apnoea was improved with low-energy-intake diets, both as a unique intervention and accompanied by exercise^(64)^. On the other hand, it should be noted that high energy intake, as well as energy-dense or nutrient-dense foods (low in fibre and high in saturated fats and sugars) and poor dietary quality are part of unhealthy routines with multiple components which may impact sleep quality such as sleep hygiene, exercise, obesity, and smoking.

As shown in Table 4, the industrialised pattern had higher intakes of the energy, sugar and fats mentioned earlier, which reinforces the associations previously documented between these consumption habits and sleep disorders.

The present study had some limitations, among them, the cross-sectional design which did not allow the establishment of causality. Furthermore, the obtention of information through self-reporting may have led to over- or underestimation of sleep disorder prevalence, and biased results within certain food group intakes by characteristics such as BMI and sex, given that certain answers may have been perceived as socially desirable^(65)^. In addition, although statistical regression models were adjusted by rural/urban area type, geographical region and physical activity level, residual confusion is possible, and the effects attributed to the industrialised dietary pattern may be further linked to factors such as urban lifestyles in more developed regions, depression or stress.

On the other hand, strengths of the present study include that our sample is nationally representative, and dietary assessment was performed using a food frequency questionnaire validated in both the adolescent and adult Mexican populations which shows an acceptable correlation between consumption, energy and macronutrients intakes estimated by 24 h recalls and food frequency questionnaire^(66)^. In addition, the dietary pattern approach encompasses the effects of the overall diet, representing a broad picture of food and nutrient consumption and offering a more accurate way of assessing disease risk than do individual food or nutrient intakes^(67,68)^. Furthermore, the association between dietary intake and sleep disorders in the Mexican population serves to generate new hypotheses which demand further research, with the final goal of improving the diet and health of Mexican adults.

## Conclusions

In conclusion, a diet high in sugar-sweetened beverages, fast foods, breads, pastries and cookies, and alcohol, coffee or tea is associated with higher odds for suffering from OSA and daytime symptoms in Mexican adults aged 20–59. It is essential to raise public awareness of the role of diet on sleep quality and health and perform further research, in order to promote evidence-based interventions aimed at achieving healthier diets in the population and preventing the negative effects, including sleep disorders, of unhealthy diets on health.

## Supplementary material

For supplementary material accompanying this paper visit http://dx.doi.org/10.1017/jns.2021.24. click here to view supplementary material
