# Supplementary material for: Dietary patterns and sleep disorders in Mexican adults from a National Health and Nutrition Survey
Source: J Nutr Sci. 2021 May 11;10:e34. doi: 10.1017/jns.2021.24 (PMC8141679; doi:10.1017/jns.2021.24)
Supplement: Supplementary file 1 [file S2048679021000240sup001.docx]

**Supplementary table 1**. Associations between dietary patterns and sleep disorders in Mexican Adults, by sex. Complete logistic regression models.

|  | **Males** | | | **Females** | | | **National** | | |
| --- | --- | --- | --- | --- | --- | --- | --- | --- | --- |
|  | OR | 95% CI | *P* value | OR | 95% CI | *P* value | OR | 95% CI | *P* value |
| **Insomnia** |  |  |  |  |  |  |  |  |  |
| Dietary pattern |  |  |  |  |  |  |  |  |  |
| Traditional | 1.00 |  |  | 1.00 |  |  | 1.00 |  |  |
| Industrialized | 1.05 | 0.58, 1.90 | 0.878 | 1.11 | 0.75, 1.63 | 0.601 | 1.08 | 0.77, 1.53 | 0.654 |
| Mixed | 0.92 | 0.52, 1.62 | 0.768 | 0.87 | 0.6, 1.24 | 0.432 | 0.87 | 0.63, 1.19 | 0.387 |
|  |  |  |  |  |  |  |  |  |  |
| Energy intake (kcal) | 1.00 | 1.00, 1.00 | 0.118 | 1.00 | 1.00, 1.00 | 0.888 | 1.00 | 1.00, 1.00 | 0.157 |
| Sex |  |  |  |  |  |  |  |  |  |
| Male |  |  |  |  |  |  | 1.00 |  |  |
| Female |  |  |  |  |  |  | 1.65 | 1.18, 2.3 | 0.003 |
| Age (years) |  |  |  |  |  |  |  |  |  |
| 20-39 | 1.00 |  |  | 1.00 |  |  | 1.00 |  |  |
| 40-59 | 1.54 | 1.03, 2.3 | 0.038 | 1.13 | 0.82, 1.56 | 0.441 | 1.24 | 0.95, 1.61 | 0.113 |
| Area type |  |  |  |  |  |  |  |  |  |
| Rural | 1.00 |  |  | 1.00 |  |  | 1.00 |  |  |
| Urban | 2.5 | 1.48, 4.2 | 0.001 | 1.08 | 0.76, 1.55 | 0.66 | 1.45 | 1.09, 1.93 | 0.011 |
| Geographical region |  |  |  |  |  |  |  |  |  |
| North | 1.00 |  |  | 1.00 |  |  | 1.00 |  |  |
| Center | 0.49 | 0.27, 0.92 | 0.025 | 1.50 | 0.97, 2.31 | 0.068 | 0.98 | 0.65, 1.49 | 0.94 |
| Mexico City | 0.51 | 0.24, 1.08 | 0.078 | 1.04 | 0.55, 1.98 | 0.904 | 0.80 | 0.47, 1.36 | 0.404 |
| South | 1.09 | 0.63, 1.88 | 0.76 | 1.12 | 0.71, 1.78 | 0.623 | 1.08 | 0.71, 1.63 | 0.734 |
| Well-being index |  |  |  |  |  |  |  |  |  |
| Tertile 1 | 1.00 |  |  | 1.00 |  |  | 1.00 |  |  |
| Tertile 2 | 1.65 | 0.97, 2.79 | 0.064 | 1.23 | 0.87, 1.73 | 0.241 | 1.39 | 1.03, 1.86 | 0.029 |
| Tertile 3 | 1.16 | 0.66, 2.05 | 0.601 | 1.31 | 0.86, 1.99 | 0.209 | 1.25 | 0.90, 1.73 | 0.189 |
| Tobacco use (lifetime) |  |  |  |  |  |  |  |  |  |
| >100 cigarettes | 1.00 |  |  | 1.00 |  |  | 1.00 |  |  |
| ≤100 cigarettes | 1.41 | 0.83, 2.41 | 0.203 | 0.73 | 0.46, 1.16 | 0.178 | 1.03 | 0.70, 1.52 | 0.885 |
| None | 1.30 | 0.74, 2.29 | 0.361 | 0.80 | 0.53, 1.22 | 0.302 | 1.04 | 0.73, 1.48 | 0.838 |
| Physical activity level |  |  |  |  |  |  |  |  |  |
| Inactive | 1.00 |  |  | 1.00 |  |  | 1.00 |  |  |
| Moderately active | 0.80 | 0.34, 1.92 | 0.620 | 0.56 | 0.32, 0.99 | 0.046 | 0.62 | 0.39, 0.99 | 0.045 |
| Active | 0.63 | 0.35, 1.15 | 0.134 | 0.89 | 0.59, 1.33 | 0.566 | 0.78 | 0.55, 1.12 | 0.184 |
| BMI | 0.98 | 0.94, 1.02 | 0.333 | 1.03 | 1.00, 1.06 | 0.067 | 1.01 | 0.99, 1.04 | 0.338 |
| **Daytime symptoms** |  |  |  |  |  |  |  |  |  |
| Dietary pattern |  |  |  |  |  |  |  |  |  |
| Traditional | 1.00 |  |  | 1.00 |  |  | 1.00 |  |  |
| Industrialized | 1.42 | 0.82, 2.46 | 0.208 | 1.51 | 1.04, 2.19 | 0.032 | 1.49 | 1.12, 1.99 | 0.007 |
| Mixed | 1.45 | 0.78, 2.70 | 0.239 | 0.75 | 0.51, 1.08 | 0.121 | 0.93 | 0.66, 1.30 | 0.665 |
|  |  |  |  |  |  |  |  |  |  |
| Energy intake (kcal) | 1.00 | 1.00, 1.00 | 0.765 | 1.00 | 1.00, 1.00 | 0.363 | 1.00 | 1.00, 1.00 | 0.617 |
| Sex |  |  |  |  |  |  |  |  |  |
| Male |  |  |  |  |  |  | 1.00 |  |  |
| Female |  |  |  |  |  |  | 2.17 | 1.51, 3.13 | <0.0001 |
| Age (years) |  |  |  |  |  |  |  |  |  |
| 20-39 | 1.00 |  |  | 1.00 |  |  | 1.00 |  |  |
| 40-59 | 1.99 | 1.19, 3.33 | 0.008 | 1.49 | 1.09, 2.02 | 0.012 | 1.64 | 1.26, 2.14 | <0.0001 |
| Area type |  |  |  |  |  |  |  |  |  |
| Rural | 1.00 |  |  | 1.00 |  |  | 1.00 |  |  |
| Urban | 1.38 | 0.85, 2.23 | 0.191 | 1.5 | 1.04, 2.17 | 0.031 | 1.46 | 1.09, 1.95 | 0.011 |
| Geographical region |  |  |  |  |  |  |  |  |  |
| North | 1.00 |  |  | 1.00 |  |  | 1.00 |  |  |
| Center | 0.90 | 0.47, 1.73 | 0.755 | 1.83 | 1.10, 3.03 | 0.02 | 1.39 | 0.93, 2.08 | 0.113 |
| Mexico City | 1.95 | 0.96, 3.97 | 0.063 | 1.77 | 0.94, 3.36 | 0.078 | 1.88 | 1.17, 3.02 | 0.010 |
| South | 1.05 | 0.59, 1.88 | 0.870 | 1.47 | 0.87, 2.49 | 0.148 | 1.28 | 0.87, 1.90 | 0.212 |
| Well-being index |  |  |  |  |  |  |  |  |  |
| Tertile 1 | 1.00 |  |  | 1.00 |  |  | 1.00 |  |  |
| Tertile 2 | 0.91 | 0.54, 1.53 | 0.728 | 1.07 | 0.79, 1.46 | 0.672 | 1.02 | 0.78, 1.35 | 0.872 |
| Tertile 3 | 1.03 | 0.58, 1.83 | 0.929 | 0.96 | 0.62, 1.48 | 0.844 | 0.98 | 0.69, 1.38 | 0.887 |
| Tobacco use (lifetime) |  |  |  |  |  |  |  |  |  |
| >100 cigarettes | 1.00 |  |  | 1.00 |  |  | 1.00 |  |  |
| ≤100 cigarettes | 1.05 | 0.64, 1.73 | 0.845 | 0.74 | 0.41, 1.33 | 0.313 | 0.87 | 0.6, 1.28 | 0.492 |
| None | 0.64 | 0.38, 1.09 | 0.104 | 0.66 | 0.43, 1.01 | 0.055 | 0.71 | 0.51, 0.98 | 0.040 |
| Physical activity level |  |  |  |  |  |  |  |  |  |
| Inactive | 1.00 |  |  | 1.00 |  |  | 1.00 |  |  |
| Moderately active | 0.54 | 0.22, 1.32 | 0.178 | 1.02 | 0.56, 1.86 | 0.945 | 0.82 | 0.51, 1.32 | 0.416 |
| Active | 0.64 | 0.37, 1.1 | 0.109 | 1.32 | 0.79, 2.20 | 0.291 | 0.97 | 0.67, 1.41 | 0.872 |
| BMI | 1.04 | 1.00, 1.08 | 0.069 | 1.02 | 0.99, 1.05 | 0.131 | 1.03 | 1.01, 1.05 | 0.015 |
| **Sleep duration<7h** |  |  |  |  |  |  |  |  |  |
| Dietary pattern |  |  |  |  |  |  |  |  |  |
| Traditional | 1.00 |  |  | 1.00 |  |  | 1.00 |  |  |
| Industrialized | 1.06 | 0.68, 1.67 | 0.797 | 1.62 | 1.02, 2.58 | 0.040 | 1.32 | 0.96, 1.80 | 0.088 |
| Mixed | 0.76 | 0.44, 1.31 | 0.323 | 1.18 | 0.8, 1.76 | 0.405 | 0.98 | 0.71, 1.36 | 0.906 |
|  |  |  |  |  |  |  |  |  |  |
| Energy intake (kcal) | 1.00 | 1.00, 1.00 | 0.965 | 1.00 | 1.00, 1.00 | 0.623 | 1.00 | 1.00, 1.00 | 0.695 |
| Sex |  |  |  |  |  |  |  |  |  |
| Male |  |  |  |  |  |  | 1.00 |  |  |
| Female |  |  |  |  |  |  | 0.84 | 0.65, 1.08 | 0.176 |
| Age (years) |  |  |  |  |  |  |  |  |  |
| 20-39 | 1.00 |  |  | 1.00 |  |  | 1.00 |  |  |
| 40-59 | 1.12 | 0.77, 1.64 | 0.546 | 1.35 | 1.01, 1.81 | 0.042 | 1.22 | 0.96, 1.55 | 0.101 |
| Area type |  |  |  |  |  |  |  |  |  |
| Rural | 1.00 |  |  | 1.00 |  |  | 1.00 |  |  |
| Urban | 1.51 | 1.07, 2.15 | 0.021 | 1.20 | 0.87, 1.65 | 0.272 | 1.35 | 1.06, 1.72 | 0.014 |
| Geographical region |  |  |  |  |  |  |  |  |  |
| North | 1.00 |  |  | 1.00 |  |  | 1.00 |  |  |
| Center | 1.22 | 0.74, 2.02 | 0.441 | 1.00 | 0.69, 1.44 | 0.997 | 1.10 | 0.80, 1.51 | 0.554 |
| Mexico City | 1.91 | 1.04, 3.49 | 0.036 | 0.80 | 0.53, 1.20 | 0.286 | 1.22 | 0.84, 1.78 | 0.297 |
| South | 1.23 | 0.72, 2.11 | 0.439 | 0.92 | 0.61, 1.41 | 0.713 | 1.07 | 0.74, 1.54 | 0.732 |
| Well-being index |  |  |  |  |  |  |  |  |  |
| Tertile 1 | 1.00 |  |  | 1.00 |  |  | 1.00 |  |  |
| Tertile 2 | 1.60 | 1.09, 2.34 | 0.016 | 1.47 | 1.06, 2.03 | 0.02 | 1.55 | 1.24, 1.94 | <0.0001 |
| Tertile 3 | 1.87 | 1.18, 2.96 | 0.008 | 1.27 | 0.84, 1.91 | 0.25 | 1.54 | 1.17, 2.02 | 0.002 |
| Tobacco use (lifetime) |  |  |  |  |  |  |  |  |  |
| >100 cigarettes | 1.00 |  |  | 1.00 |  |  | 1.00 |  |  |
| ≤100 cigarettes | 0.94 | 0.63, 1.38 | 0.735 | 1.00 | 0.58, 1.73 | 0.999 | 0.94 | 0.70, 1.26 | 0.66 |
| None | 0.73 | 0.47, 1.14 | 0.167 | 0.79 | 0.49, 1.27 | 0.323 | 0.76 | 0.57, 1.02 | 0.063 |
| Physical activity level |  |  |  |  |  |  |  |  |  |
| Inactive | 1.00 |  |  | 1.00 |  |  | 1.00 |  |  |
| Moderately active | 0.63 | 0.28, 1.44 | 0.274 | 0.67 | 0.33, 1.37 | 0.271 | 0.66 | 0.39, 1.12 | 0.121 |
| Active | 1.11 | 0.69, 1.81 | 0.660 | 1.13 | 0.66, 1.93 | 0.666 | 1.12 | 0.79, 1.58 | 0.528 |
| BMI | 1.04 | 1.01, 1.08 | 0.023 | 1.04 | 1.01, 1.07 | 0.014 | 1.04 | 1.01, 1.06 | 0.001 |
| **Obstructive Sleep Apnea** | |  |  |  |  |  |  |  |  |
| Dietary pattern |  |  |  |  |  |  |  |  |  |
| Traditional | 1.00 |  |  | 1.00 |  |  | 1.00 |  |  |
| Industrialized | 1.87 | 1.24, 2.81 | 0.003 | 1.41 | 0.92, 2.14 | 0.113 | 1.63 | 1.21, 2.19 | 0.001 |
| Mixed | 1.31 | 0.82, 2.11 | 0.253 | 1.00 | 0.65, 1.54 | 0.992 | 1.14 | 0.83, 1.55 | 0.424 |
|  |  |  |  |  |  |  |  |  |  |
| Energy intake (kcal) | 1.00 | 1.00, 1.00 | 0.412 | 1.00 | 1.00, 1.00 | 0.542 | 1.00 | 1.00, 1.00 | 0.392 |
| Sex |  |  |  |  |  |  |  |  |  |
| Male |  |  |  |  |  |  | 1.00 |  |  |
| Female |  |  |  |  |  |  | 1.25 | 0.93, 1.68 | 0.140 |
| Age (years) |  |  |  |  |  |  |  |  |  |
| 20-39 | 1.00 |  |  | 1.00 |  |  | 1.00 |  |  |
| 40-59 | 2.97 | 2.07, 4.27 | <0.0001 | 2.48 | 1.76, 3.5 | <0.0001 | 2.67 | 2.08, 3.41 | <0.0001 |
| Area type |  |  |  |  |  |  |  |  |  |
| Rural | 1.00 |  |  | 1.00 |  |  | 1.00 |  |  |
| Urban | 1.15 | 0.79, 1.67 | 0.472 | 1.24 | 0.82, 1.89 | 0.310 | 1.22 | 0.93, 1.6 | 0.153 |
| Geographical region |  |  |  |  |  |  |  |  |  |
| North | 1.00 |  |  | 1.00 |  |  | 1.00 |  |  |
| Center | 0.68 | 0.38, 1.20 | 0.181 | 1.21 | 0.81, 1.83 | 0.353 | 0.91 | 0.63, 1.30 | 0.596 |
| Mexico City | 1.25 | 0.65, 2.42 | 0.505 | 1.57 | 0.85, 2.91 | 0.149 | 1.40 | 0.90, 2.19 | 0.139 |
| South | 1.23 | 0.68, 2.23 | 0.496 | 1.16 | 0.75, 1.80 | 0.502 | 1.15 | 0.79, 1.68 | 0.470 |
| Well-being index |  |  |  |  |  |  |  |  |  |
| Tertile 1 | 1.00 |  |  | 1.00 |  |  | 1.00 |  |  |
| Tertile 2 | 1.32 | 0.86, 2.03 | 0.208 | 1.05 | 0.72, 1.52 | 0.802 | 1.16 | 0.88, 1.53 | 0.289 |
| Tertile 3 | 1.10 | 0.69, 1.74 | 0.698 | 0.92 | 0.63, 1.35 | 0.676 | 0.99 | 0.73, 1.34 | 0.951 |
| Tobacco use (lifetime) |  |  |  |  |  |  |  |  |  |
| >100 cigarettes | 1.00 |  |  | 1.00 |  |  | 1.00 |  |  |
| ≤100 cigarettes | 0.87 | 0.57, 1.33 | 0.522 | 0.69 | 0.41, 1.18 | 0.180 | 0.80 | 0.56, 1.14 | 0.212 |
| None | 0.81 | 0.53, 1.24 | 0.333 | 0.62 | 0.38, 1.01 | 0.056 | 0.72 | 0.52, 1.01 | 0.055 |
| Physical activity level |  |  |  |  |  |  |  |  |  |
| Inactive | 1.00 |  |  | 1.00 |  |  | 1.00 |  |  |
| Moderately active | 0.36 | 0.16, 0.78 | 0.010 | 0.55 | 0.32, 0.94 | 0.030 | 0.45 | 0.29, 0.70 | <0.0001 |
| Active | 0.35 | 0.22, 0.56 | <0.0001 | 0.71 | 0.46, 1.11 | 0.137 | 0.52 | 0.37, 0.73 | <0.0001 |
